# Total Glucosides of Paeony Promote Intestinal Motility in Slow Transit Constipation Rats through Amelioration of Interstitial Cells of Cajal

**DOI:** 10.1371/journal.pone.0160398

**Published:** 2016-08-01

**Authors:** Feiye Zhu, Shan Xu, Yongsheng Zhang, Fangming Chen, Jinjun Ji, Guanqun Xie

**Affiliations:** 1 Center of Analysis and Testing, Zhejiang Chinese Medical University, Hangzhou, China; 2 College of Basic Medical Science, Zhejiang Chinese Medical University, Hangzhou, China; 3 Library of Zhejiang Chinese Medical University, Hangzhou, China; 4 Laboratory animal center, Zhejiang Chinese Medical University, Hangzhou, China; Universita degli Studi di Napoli Federico II, ITALY

## Abstract

**Objectives:**

Using an atropine-diphenoxylate-induced slow transit constipation (STC) model, this study explored the effects of the total glucosides of paeony (TGP) in the treatment of STC and the possible mechanisms.

**Study Design:**

A prospective experimental animal study.

**Methods:**

The constipation model was set up in rats with an oral gavage of atropine-diphenoxylate and then treated with the TGP. The volume and moisture content of the faeces were observed and the intestinal kinetic power was evaluated. Meanwhile, the colorimetric method and enzyme linked immunosorbent assay (ELISA) were employed to determine the changes of nitric oxide (NO), nitric oxide synthase (NOS), vasoative intestinal peptide (VIP) and the P substance (SP) in the serum, respectively. The protein expressions of c-kit and stem cell factor (SCF) were assessed by immunohistochemical analysis and western blot, respectively, and the mRNA level of c-kit was measured by a reverse transcription polymerase chain reaction (RT-PCR).

**Results:**

The TGP attenuated STC responses in terms of an increase in the fecal volume and moisture content, an enhancement of intestinal transit rate and the reduction of NO, NOS and VIP in the serum. In addition, the c-kit, a labeling of interstitial cells of Cajal (ICC) increased at both protein and mRNA levels. SCF, which serves as a ligand of c-kit also increased at protein level.

**Conclusion:**

The analysis of our data indicated that the TGP could obviously attenuate STC through improving the function of ICC and blocking the inhibitory neurotransmitters such as NO, NOS and VIP.

## Introduction

Slow transit constipation (STC), an intractable constipation, is usually characterized by a heavily delayed colonic transit up to true colonic inertia [1]. This is a very prevalent motility problem, but its aetiology has not been elucidated as yet [2]. Even so, compelling evidence has revealed that interstitial cells of Cajal (ICC) contribute to pathogenesis of STC [3–5].

ICC is found between the nerve endings and smooth muscle cells in the gastrointestinal (GI) tract. It is recognized not only as pacemaker cells for gastrointestinal movement but also as mediators of neuromuscular transmission [6]. Studies have demonstrated that ICC density in the colon of patients with STC significantly decreased compared with those of normal patients [7,8]. Therefore, a decreased number of ICC might lead to absence of slow wave activity, thereby affecting the contractile response and causing delayed transit in STC patients. In this regards, amelioration of ICC function using drugs may be essential to treat STC.

Total glucosides of paeony (TGP) is extracted from the root of Paeonia lactiflora pall, which has been used for cramp and pain for over 1,500 years in traditional Chinese medicine. TGP, which contains paconiflorin (>90%) and other components such as hydroxyl-paconiflorin, paeonin, albiflorin, benzoylpaeoniflorin and so on, was approved by State Food and Drug Administration (SFDA) of China (Approval No. H20055058) to enter the market as a disease-modifying drug for rheumatoid arthritis(RA) in 1998[9].A series of previous studies have demonstrated the most adverse events of TGP were gastrointestinal tract disturbances, mostly mild diarrhea, and no adverse events following hepatic, renal, or hematological tests were reported[10–12]. However, its side-effect of leading diarrhea caught our attention. Whether TGP is involved in mediating intestinal motility remains uncertain. This study is important for investigating the intestinal promotion function of TGP and its functional effect on ICC.

## Materials and Methods

### Animals

The experimental protocol was approved by the Animal Welfare Committee of Zhejiang Chinese Medical University, Hangzhou, China (SYXK2008-0115). The animal housing facility is a barrier housing facility, conforming to the national standard of China (Laboratory Animal-Requirements of Environment and Housing Facilities) (GB14925-2001). Female Munich-Wistar rats, weighing 160±20g, were obtained from Laboratory Animal Research Center of Zhejiang Chinese Medical University, China.

### Drugs and Reagents

The Rat vasoative intestinal peptide (VIP) enzyme linked immunosorbent assay (ELISA) kit and the P substance (SP) ELISA kit were bought from Cusabio Biotech Co., Ltd (Wuhan, Hubei, China). The nitric oxide (NO) and the nitric oxide synthase (NOS) kit were purchased from Nanjing Jiancheng Bioengineering Institute (Nanjing, Jiangsu, China). In this study, primary antibodies used for western blot included rabbit anti-SCF and anti-β-actin antibodies (Santa Cruz Biotechnology, CA, USA). cDNA synthesis Kit (TaKaRa Bio, Siga, Japan) was used for reverse transcription. C-kit andβ-actin primers used for reverse transcription polymerase chain reaction (RT-PCR) were designed and synthesized by Sangon Biotech (Shanghai) Co., Ltd (Shanghai, China). Compound diphenoxylate was purchased from Changzhou Kangpu Pharmaceutical Co., Ltd, Changzhou, China (lot number: 1010004). TGP capsules were purchased from Ningbo Liwah Pharmaceutical Co., Ltd, Ningbo, China (lot number: 110705). The extract was obtained with the following procedure [13]. The root of white peony material was extracted with 70% aqueous ethanol, 2 h each time. Then, the extracts were combined and evaporated to dryness under reduced pressure. The residue obtained from the combined extract was dissolved with water. After filtration, the aqueous solution was extracted three times with ethyl acetate successively each time, and then evaporated to obtain the crude extract. Then the ethyl acetate extract was chromatographed on polystyrene resin with water and 20% ethanol, respectively. Portions of 20% ethanol were collected and evaporated to get the final extract. According to the information supplied by the medical corporation and the results of HPLC analysis, the TGP mainly contained paeoniflorin(90.42%), hydroxyl-paeoniflorin(0.24%), paeonin(0.93%), albiflorin (0.14%), benzoylpaeoniflorin(3.44%), and some trace substances(about 4.8% total). The amount of each substance was calculated based on the peak area of HPLC.

### Experimental Design

The atropine-diphenoxylate was used to induce constipation in rats according to previous reports [14,15]. A total of 30 rats were randomly divided into 3 groups (n = 10 per group): normal control group (NC), model control group (MC) and group of STC rats treated with TGP (MC+ TGP). NC: treated with normal saline (10ml/kg); MC: treated with atropine (0.1mg/kg)–diphenoxylate (10mg/kg); MC+ TGP: treated with atropine (0.1mg/kg)—diphenoxylate(10mg/kg) and TGP (0.18g/kg).

Except the NC rats, others were orally administered by atropine-diphenoxylate once daily for 14 consecutive days to induce constipation. The TGP was suspended in water and was orally administered once daily from day 15 to day 28 in the MC+TGP group, while normal saline was given gastric gavage at 1 ml/100g in the NC group and MC group.

### Observation of the Fecal Volume and Moisture Content

The animal faeces were collected and counted during the experimental process. The wet weight (A) of faeces was recorded immediately after collection, and dry weight (B) of faeces was recorded after they were dried in drying oven for 3h. The moisture content was calculated as follows: the fecal moisture percentage (%) = (A-B)/A*100%.

### Blood Sample and Tissue Collection

After 14 days of treatment, rats were fasted for 12h but allowed free access to water. Each rat was administered with 0.2 ml of standard charcoal meal (5% activated charcoal suspended in 10% gum acacia) orally. Rats were then anesthetized 30 min later by intraperitoneal injection of sodium pentobarbital (50 mg/kg) and placed on a temperature-regulated table. Blood samples were collected and centrifuged at 3500 rpm for 15 min to obtain serum. The intestine was carefully inspected and the distance traversed using the charcoal meal from the pylorus was measured for all the groups. The colon was removed immediately and flushed with normal saline at 4°C, and then sectioned into two pieces. One fragment was fixed in 10% formalin and processed in paraffin for subsequent immunohistochemical studies, while the other was stored at -80°C until they were assayed.

### Evaluation of the Intestinal Kinetic Power

The intestinal transit rate was calculated as follows: intestinal transit rate (%) = *A*/*B*×100% (*A*: length among the musculus sphincter pylori and the end of charcoal stained intestine; *B*: whole length of the intestine (distance from pylorus to rectum).)

### Assays of Blood Sample

The levels of NO and NOS were determined by colorimetric method using commercial reagents. The VIP and SP concentrations in the serum were estimated by ELISA using commercially available kits.

### Immunohistochemical Analysis

Unstained 5μm sections were cut from paraffin blocks for immunohistochemical (IHC) analysis. The sections were stained with rabbit anti-c-kit (1:100) at 4°C overnight. The secondary antibody and avidin-biotin peroxidase complex method were used according to the manufacture’s instructions. An immunoglobulin-negative control was used to eliminate nonspecific binding. Expressions of c-kit were determined by the semi-quantitative method. Using Carl Zeiss imaging system (Carl Zeiss Imaging Systems, Germany), the area of stained cells were measured under 40×objective lens of each slice for semi-quantitative analysis.

### RT-PCR

Total RNA was extracted according to the manufacture’s protocol using TRIzol Reagent (Invitrogen, USA). The total RNA (1μg) was reverse-transcripted into cDNA using SuperScript II reverse transcriptase. The operations were done strictly in accordance with the manufacturer’s protocol. The expression level of c-kit was analyzed by semi-quantitative RT-PCR with β-actin as internal control. The primer sequences were designed as follows: c-kit forward 5´-AGATGACGAGCTGGCTCTGGA-3´and reverse 5´-CTGGCTGCCAA ATCTCTGTGAA-3´;β-actin forward 5´-ACACTGTGCCCATCTACG-3´ and reverse 5´-CAGGATTCCATACCCAAG-3´. After 30 cycles of amplification, 5μl of amplification product was used for gel electrophoresis.

The software program Gel-Pro Analyzer (BIO-RAD, USA) was used for quantitative analysis of the stained gels. The integrated optical density (IOD) of the bands on digitized images was measured. All RT-PCR reactions were done 3 times for each sample. C-kit gene expression was expressed as the ratio of c-kit overβ-actin. The ratio between the PCR amplified gene and the amplified standard was obtained for each sample.

### Western Blot Analysis

Colon samples were homogenized and lysed in SDS-PAGE sample buffer, boiled, centrifuged and supernatant recovered. Samples were run on 10% SDS polyacrylamide gels, electroblotted onto nitrocellulose membranes, incubated with blocking buffer for 1h. Immunoblotting was assayed using anti-SCF (1:200) antibodies. Anti-β-actin (1:200) was used as loading control. The detection and analysis were done by Odyssey Infrared Imaging System (Gene Company Ltd., Hongkong, China).

### Statistical Analysis

Measurement data were expressed as mean±SEM and analyzed by SPSS 16.0 software. Significant differences between the experimental groups were analyzed by the Student’s *t* test or Mann-Whitney *U* test when appropriate. *P* <0.05 was accepted as statistically significant.

## Results

### The Intervention with TGP Partially Recovered Intestinal Function

**Fig 1:**
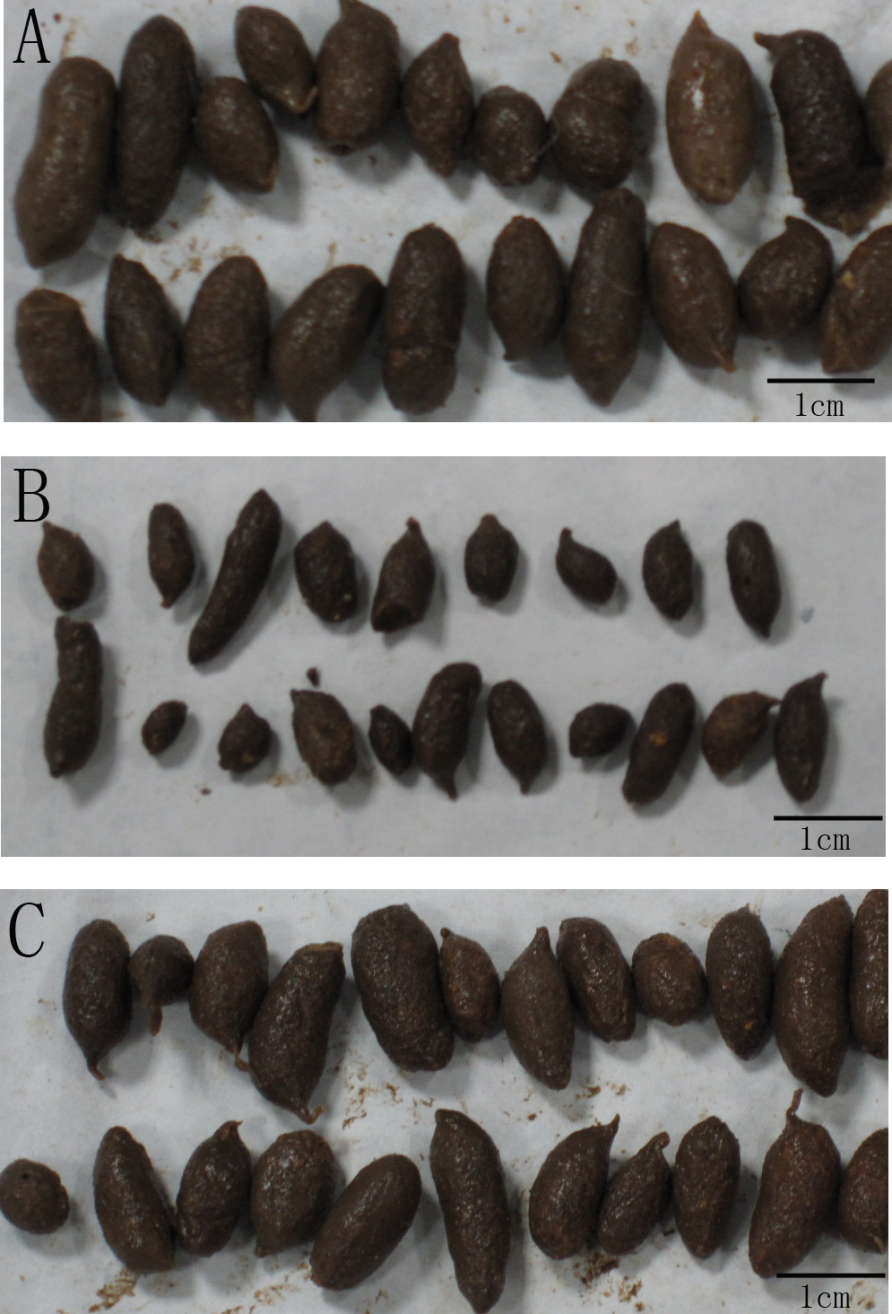
The effect of TGP on the faeces in rat models of STC. The characters of faeces were compared between NC (A), MC (B) and MC+TGP (C) groups.

**Fig 2:**
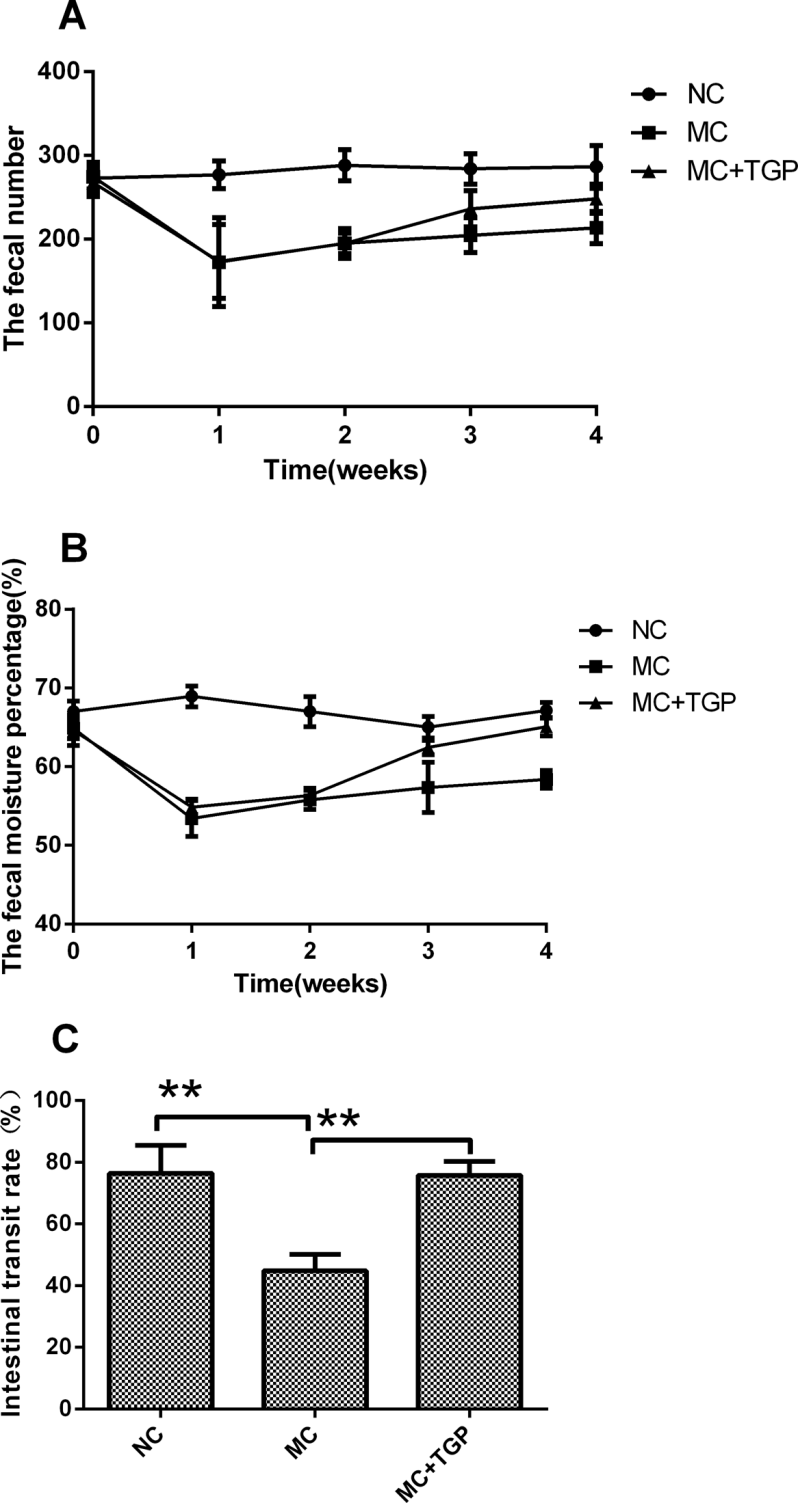
The effect of TGP on the intestinal function in rat models of STC. The fecal number (A) and moisture content (B) decreased in the 1st and 2nd week, while they increased after TGP treatment. Each dot represents the mean±SEM, n = 10(each of the three groups). The intestinal transit rate(C) was compared among NC, MC and MC+TGP groups. Each bar represents the mean±SEM, n = 10 (each of the three groups). * *p*<0.05, **p<0.01 as conducted.

Compared with normal faeces, the faeces defecated by rats in the MC group were dry, small, hard and without burnish (Fig 1A and 1B). This condition ameliorated after treatment by TGP (Fig 1C). The number and moisture content of faeces decreased after oral gavage of atropine- diphenoxylate, whereas they increased when treated by TGP (Fig 2A and 2B). Fig 2C showed the intestinal transit rate from 3 groups, respectively. The intestinal transit rate in MC group was statistically significantly lower than that in NC group (*P*<0.01), whereas the rate in the treatment group was higher than that in MC group (*P*<0.01).

### TGP Treatment Reduced the Inhibitory Neurotransmitters

**Fig 3:**
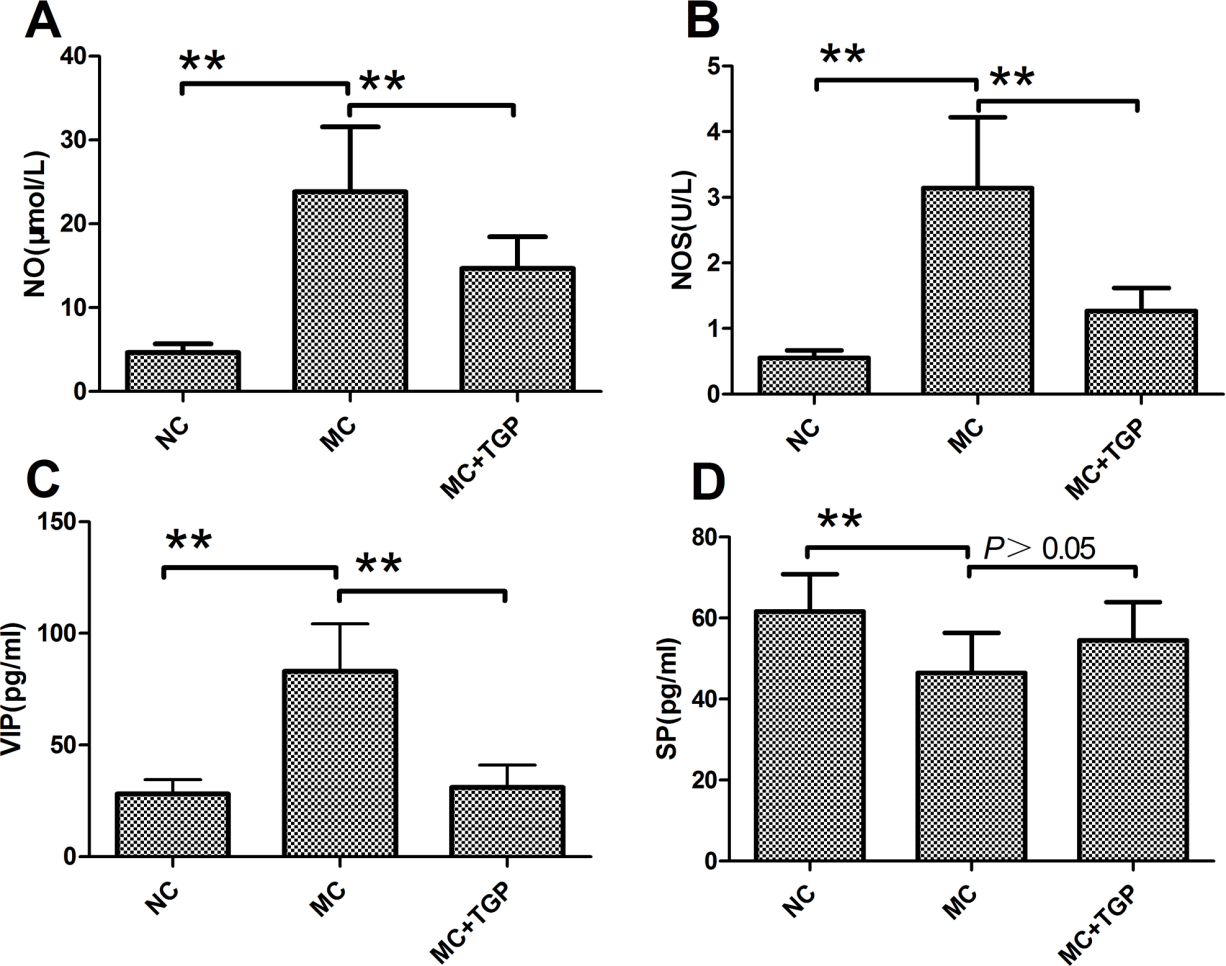
TGP treatment reduced the inhibitory neurotransmitter. The levels of NO (A), NOS (B) and VIP (C) in the MC group increased compared with that in the NC group, while they decreased after TGP treatment. The level of SP (D) decreased in the MC group, but it did not obviously increase in the MC+TGP group. Each bar represents the mean±SEM, n = 10 (each of the three groups). * *p*<0.05, **p<0.01 as conducted.

The values of NO and NOS of the MC group rats were increased when compared with the normal rats (*P*<0.01, respectively). Elevated NO and NOS was significantly attenuated by TGP (*P*<0.01, respectively) (Fig 3A and 3B). The serum levels of VIP obtained from MC group rats were increased significantly compared with those from NC group (*P*<0.01). Administration of TGP attenuated the increased VIP (*P*<0.01) (Fig 3C). Compared with normal rats, constipation rats attenuated SP levels(*P*<0.01), and administration of TGP could not change the levels of SP (*P*>0.05) (Fig 3D).

### TGP Ameliorated the Function of ICC

**Fig 4:**
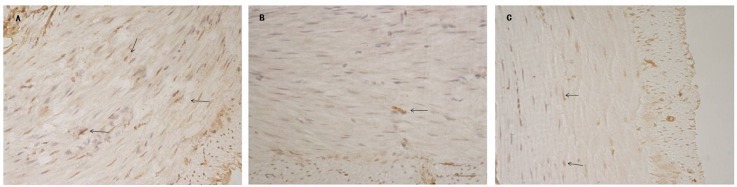
IHC determination of c-kit in the intestinal mucosa. Original magnification, ×40. Numerous positive cells are displayed in the intestinal mucosa in the rats of NC and MC+TGP groups (A, C). Only few positive cells are observed in the intestinal mucosa of the STC animals (B). Arrow: positive cells.

**Fig 5:**
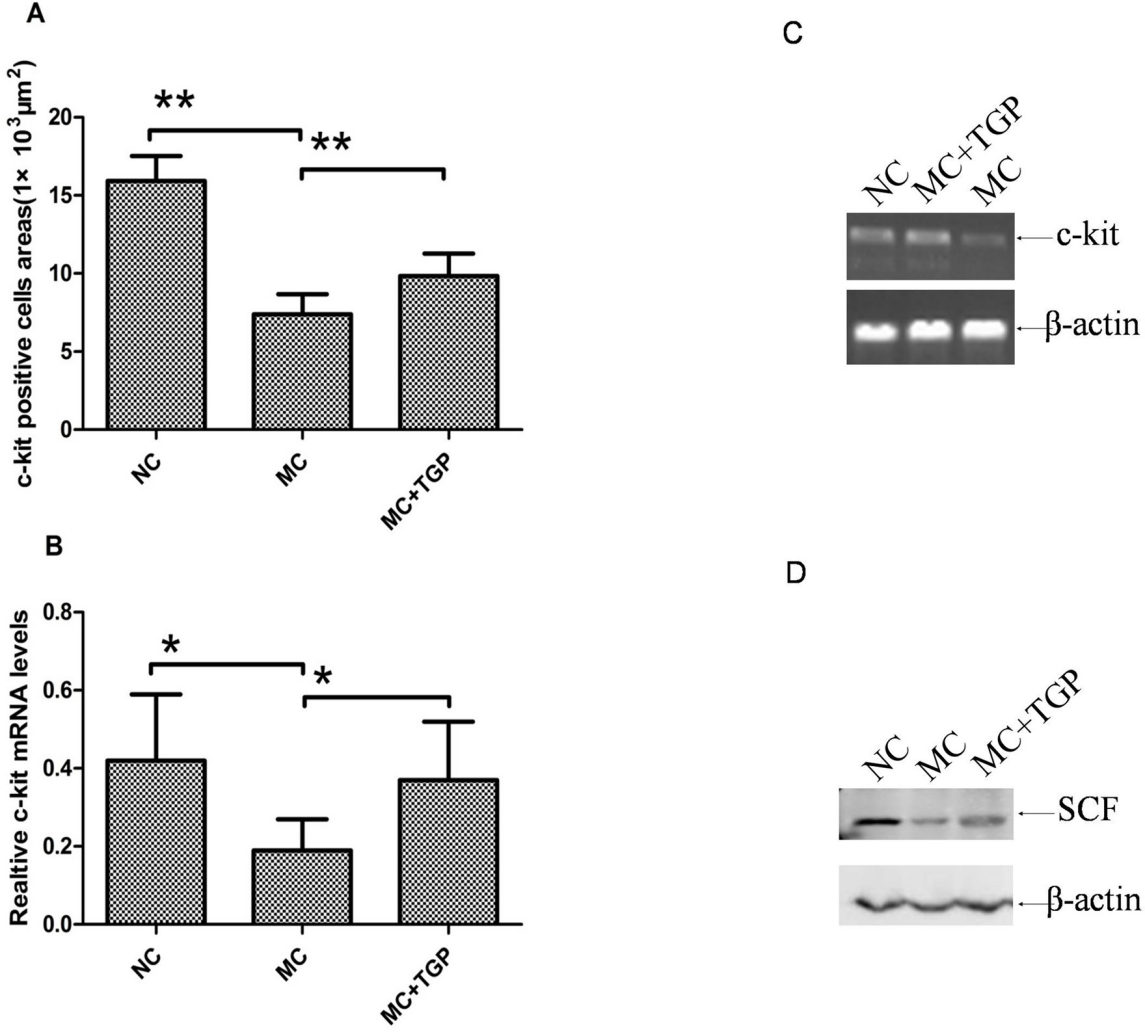
TGP ameliorated the function of ICC. The protein expression (A) and mRNA levels (B, C) of c-kit in the MC group decreased compared with that in the NC group, while they increased after TGP treatment. The protein expression of SCF (D) decreased in the MC group, while it increased in the MC+TGP group. Each bar represents the mean±SEM, (A) n = 10 (each of the three groups), (B, D) n = 6 (each of the three groups). * *p*<0.05, **p<0.01 as conducted.

To assess the change of ICC in intestinal mucosa, immunohistochemical analysis and RT-PCR were done to investigate expression levels of protein and mRNA of c-kit. The results revealed that fewer c-kit positive cells were observed in the intestinal mucosa of the STC animals than those in the normal rats (Fig 4A and 4B). As shown in Fig 4C, c-kit positive signs were widely distributed in intestinal mucosa of STC animals with treatment of TGP. The positive cells areas also showed that c-kit expression levels in intestinal mucosa of constipation rats were reduced(*P*<0.01). The decreased c-kit in constipation rats was significantly increased by TGP treatment (*P*<0.01) (Fig 5A). The results showed the mRNA levels of c-kit in MC group was statistically significantly decreased compared with that in the NC group (*P*<0.05), whereas the mRNA levels in TGP group was higher than that in MC group(*P*<0.05) (Fig 5B and 5C). We also examined the expression of SCF, a ligand of c-kit. The protein expression of SCF in the MC group rats was statistically significantly lower than that in the NC group, whereas treatment with TGP increased SCF protein expression (Fig 5D).

## Discussion

STC is a common health problem which usually attribute to abnormal motor function of the large bowel such as decreased colonic propulsive function, overall reduced electrical or motor activity of the large bowel, alterations of rectosigmoid contractile activity, and abnormal response to food ingestion [16–18].Studies have shown that the intestinal transit rate of atropine-diphenoxylate induced rats was significantly reduced compared with that of normal animals, which indicated the successful establishment of the animal model of STC. Our study also proved significant improvement of colonic transit after TGP treatment in STC rats. This result suggested that TGP showed promotion function on intestinal motility.

The motor activity of the GI tract is a complex process involving multiple cell types such as enteric neurons, ICC and smooth muscle cells. Although ICC that transduces inputs from enteric motor neurons and generates intrinsic electrical rhythmicity is a minor component of the tunica muscularis of the GI tract, it plays a very significant physiological role in intestinal motility [19]. Several neurotransmitter receptors have been identified in the ICC. Some of them are excitatory (SP) and others inhibitory (VIP and NO) transmitters [20]. ICC accepts neurotransmitters via these receptors and then transfers them to neighboring smooth muscle cells via gap junctions [6]. Previous studies showed that neurotransmitters such as NO, VIP and SP were crucial in the STC [21]. Excessive production of NO and VIP may inhibit colonic motility in STC colon. Low numbers of substance P-immunoreactive (SP-IR) nerve fibers have been found in colonic circularmuscle from STC patients [22]. This study found that the serum levels of NO, NOS and VIP in the model group were increased and SP was decreased. In the TGP group, the serum levels of NO, NOS and VIP were reduced compared with those in the model group. These data suggested that TGP played a part in intestinal motility, possibly by decreasing the inhibitory neurotransmitters.

Many ICC express c-kit, which is a membrane receptor with tyrosine kinase activity [23]. C-kit acts as a receptor for stem cell factor (SCF), which serves as its ligand. In its normal state, c-kit is present as a monomer in the cell membrane. This will activate intracellular signaling pathways that are pivotal for normal cellular growth and development [24]. Neither antibodies to kit, nor SCF, nor tyrosine kinase inhibitors could inhibit the proliferation of these cells, while mature ICC were greatly affected [25]. The c-kit signal pathway may be essential in ICC reduction in STC because the expression of c-kit mRNA and c-kit protein was significantly decreased in the colon of STC [26]. In our experiment, the expression of c-kit mRNA and c-kit protein and SCF protein were down-regulated in the STC rats. As these decreased expressions were up-regulated by TGP, TGP had obvious protective effect on ICC.

## Conclusions

In conclusion, TGP promoted the intestinal motility by improving the function of ICC and regulating neurotransmitters. The present study provided scientific evidence that TGP is a possible agent for the treatment of constipation.
